# Patients undergoing periodontal procedures commonly use dietary supplements: A consideration in the design of intervention trials

**DOI:** 10.1002/cre2.328

**Published:** 2020-09-21

**Authors:** Jennifer R. Beaudette, Peter C. Fritz, Philip J. Sullivan, Assunta Piccini, Wendy E. Ward

**Affiliations:** ^1^ Department of Kinesiology Brock University St. Catharines Ontario Canada; ^2^ Periodontal Wellness & Implant Surgery Fonthill Ontario Canada; ^3^ Center for Bone and Muscle Health, Brock University, St. Catharines Ontario Canada

**Keywords:** graft, implant, nutritional supplements, periodontal surgery

## Abstract

**Objective:**

Diet and dietary supplement use are associated with periodontal health while a cause and effect relationship is less clear. Although associations with specific nutrients and supplements suggest a potential benefit to healing of periodontal tissues after periodontal procedures, this study determined if patients undergoing periodontal surgery currently take dietary supplements to gage whether patients may accept use of such supplements as a potential intervention.

**Materials and methods:**

Patients who were undergoing implant placement or soft tissue graft surgery completed a questionnaire indicating any dietary supplements they consumed. Patient demographics, such as age, sex, and smoking status, were gathered from patients' charting records.

**Results:**

Data on dietary supplement usage were collected from 221 patients. More than half (64.7%) the population surveyed reported using one or more dietary supplements. The most commonly used dietary supplements were vitamin D (31%), multivitamin (28%), and B‐complex (17.2%). Females were more likely to be taking calcium, vitamin B12, and magnesium than males. Adults, aged 51 years and older, were more likely to be taking dietary supplements than their younger counterparts. They were also more likely to be taking four or more supplements than those under the age of 50 years. There was no association between supplement use and sex, but when the number of different supplements being used was assessed, females were more likely than males to be taking four or more different supplements.

**Conclusions:**

The majority of the study population is already taking dietary supplements as part of their routine. Based on this study, future studies to determine if supplement usage, potentially at levels higher than current levels of intake, can be used to maintain or promote periodontal health seem highly feasible.

## 1 INTRODUCTION

Periodontal disease has a prevalence of 46% among US adults (Eke et al., 2015), and understanding the development, and mitigating factors of periodontal disease is important to improve overall health. Diet may be one of these factors. Many nutrients are associated with wound healing, including protein, vitamin C, and magnesium, which are known to support collagen synthesis (Lau, Johnston, Fritz, & Ward, 2013; Li, Tang, Lin, & Xie, 2018; Stechmiller, 2010). Higher intakes of fruits and vegetables, as well as nutrients with potential antioxidant and anti‐inflammatory activity (vitamin C, α‐tocopherol, β‐carotene, EPA, and DHA), have been associated with improved healing following sanative therapy among nonsmokers (Dodington, Fritz, Sullivan, & Ward, 2015). Vitamin B complex has also been of interest for its potential role in improved periodontal healing after access flap surgery (Neiva, al‐Shammari, Nociti Jr, Soehren, & Wang, 2005). To date, there is little evidence available to indicate if these nutrients or dietary changes are also associated with improved healing following implant placement or soft tissue graft surgery; although better healing following these surgeries will improve the patient outcome. For example, following third molar extraction, patients who consumed a fruit and vegetable supplement—effectively increasing levels of vitamin C, α‐tocopherol, α‐carotene, and β‐carotene—reported less severe post‐surgery symptoms, such as less pain (Gorecki et al., 2018). Knowing a relationship between a healthful diet and periodontal health exists, there is increasing interest in the effects of dietary supplements on periodontal health. This includes exploring if specific nutrients—either at dietary or supplemental levels—may be associated with better healing after periodontal procedures aimed at reducing periodontal inflammation. Supplemental levels of nutrients, rather than dietary levels, may be needed to have a measurable effect on healing.

Before planning such intervention studies with dietary supplements, it is important to determine regular usage among patients as this can serve as a surrogate measure of acceptability, and to also determine if there are specific segments of the patient population that tend to use supplements more than others. The current study specifically studied patients who received a dental implant placement or soft tissue graft. This is a unique group of patients because they are undergoing procedures where supplements could potentially enhance healing and, by extension, may potentially reduce pain experienced with such procedures. The objective of this study was to determine the use of dietary supplements among patients undergoing periodontal surgery, specifically a dental implant placement or soft tissue graft, to shape future studies that investigate the role of such supplements in supporting healing post‐surgery.

## 2 METHODS

### 2.1 Recruitment

A convenience sample of 221 patients attending a clinic in Ontario, Canada, for soft tissue graft surgery or implant placement surgery was used. Male and female patients who were 19 years and older were invited to participate. This study was approved by the research ethics board at Brock University (13‐172‐WARD) and was registered at clinicaltrial.gov as NCT03064178.

### 2.2 Data collection

Patients completed a comprehensive questionnaire that included 32 commonly used vitamins, minerals, and other nutritional supplements to assess their usual usage of such products. There was also space for patients to add any additional supplements they were using that had not been listed. The self‐administered questionnaire was completed when patients came for their initial visit to the clinic and was kept as part of their patient record. Patient data, such as age, sex, and smoking status, were also collected. These data were collected as part of a larger study that reported different factors affecting pain perception among patients undergoing periodontal surgery (Beaudette, Fritz, Sullivan, Piccini, & Ward, 2018). The use of dietary supplements was recorded because many supplements have physiological activities, such as anti‐inflammatory, and antioxidant effects, that may modulate healing, and attenuate pain experienced with periodontal procedures.

### 2.3 Statistical analysis

Frequency of use was tabulated for each supplement. Because of the categorical nature of the variables, chi‐square analyses were performed to measure the associations between supplement use and patient characteristics, such as age, and sex. IBM SPSS Statistics, version 24, was used to perform statistical analyses.

## 3 RESULTS

### 3.1 Study population characteristics

Supplement use data were collected from 221 patients between May 2014 and March 2016 (Table 1). Of these patients, 52% were receiving soft tissue grafts and 48% were undergoing dental implant placement. Patients undergoing either a connective tissue graft or a mucogingival graft were categorized as having a soft tissue graft. There were 141 females (63.8%) and 80 males (36.2%) included in the sample. Patients were grouped by age using the same age cut‐offs used for the dietary reference intakes. Patient age ranged from 19 to 80 years of age with a mean age of 51 years. Of the 221 patients recruited, 143 (64.7%) reported using one or more supplements. Nonsmokers constituted 94.0% of the study population.

**TABLE 1:** Study population characteristics

		Frequency	Percent (%)
Age in years	19–30	30	13.6
	31–50	58	26.2
	51–70	118	53.4
	>70	15	6.8
Sex	Male	80	36.2
	Female	141	63.8
Smoking status	Nonsmoker	204	94.0
	Smoker	13	6.0
Surgery	Graft	115	52.0
	Implant	106	48.0
Supplement use	Yes	143	64.7
	No	78	35.3

### 3.2 Supplement use

**FIGURE 1:**
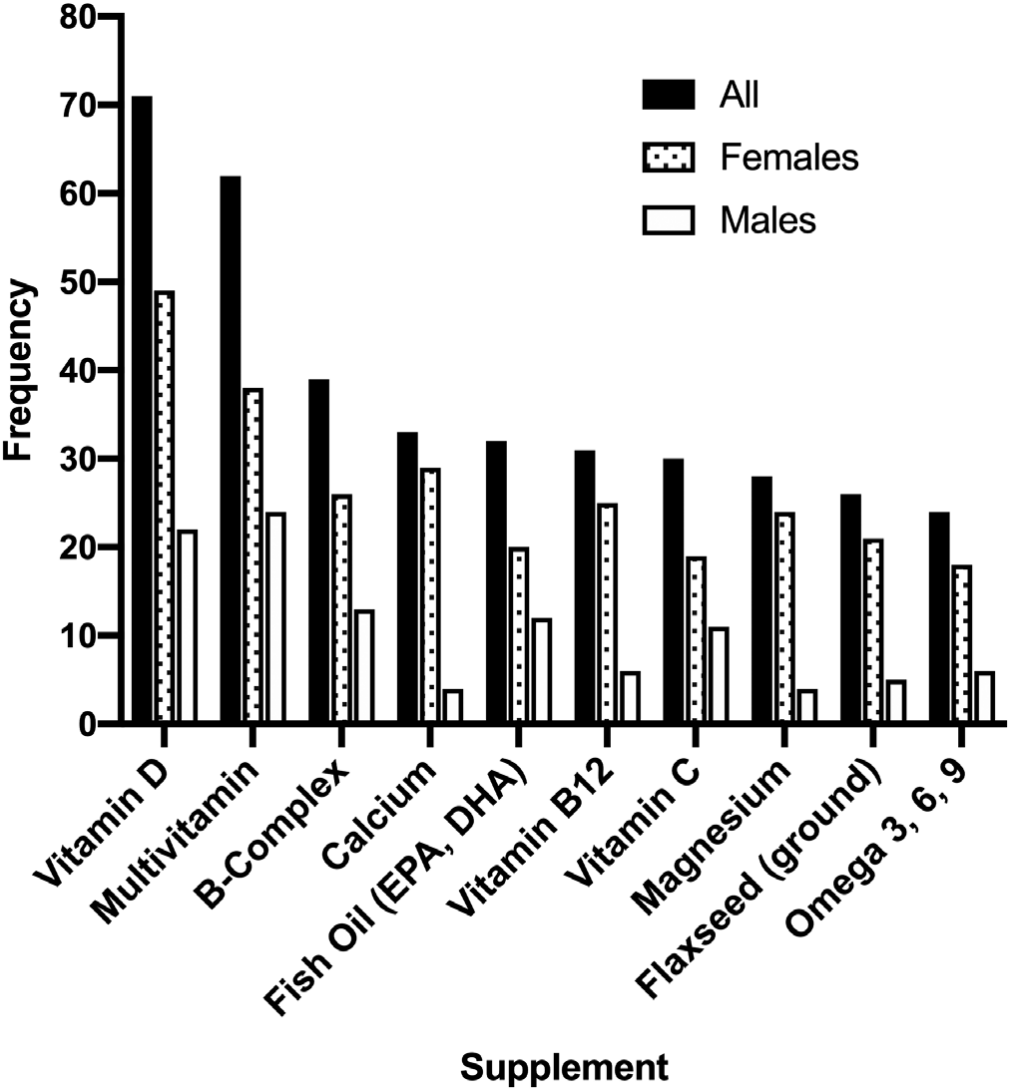
The top 10 most frequently used supplements of the entire sample of patients and in males or females only

In the sample of patients, 143 (64.7%) were supplement users; leaving 78 (35.3%) as nonusers (Figure 1; Table 2). Many patients were taking multiple dietary supplements. Of the 221 patients, 53 (24.0%) used four or more different supplements while 90 (40.7%) used one to three different supplements. The 10 most frequently supplements are shown in Figure 1 and Table 2. Vitamin D was most frequently used, with 71 patients reporting use. This represented 32.1% of the total sample and almost half of all the supplement users (49.7%). Following vitamin D, a multivitamin was the most commonly used supplement (62 patients), while the third most popular supplement was vitamin B complex (39 patients).

**TABLE 2:** Frequency of use of top 10 most frequently used dietary supplements

Supplement	Frequency	% of all patients	Frequency males, *n*	% males	Frequency females, *n*	% females	Males vs. females *p* value^a^
Vitamin D	71	32.1%	22	27.5%	49	34.8%	.269
Multivitamin	62	28.1%	24	30.0%	38	27.0%	.630
B complex	39	17.6%	13	16.3%	26	18.4%	.683
Calcium	33	14.9%	4	5.0%	29	20.6%	**.002**
Fish oil^b^	32	14.5%	12	15.0%	20	14.2%	.869
Vitamin B12	31	14.0%	6	7.6%	25	17.8%	**.035**
Vitamin C	30	13.6%	11	13.8%	19	13.5%	.955
Magnesium	28	12.7%	4	5.0%	24	17.0%	**.010**
Flaxseed (ground)	26	11.8%	5	6.6%	21	14.9%	.056
Omega 3, 6, 9	24	10.9%	6	7.6%	18	12.8%	.228

^a^ Bolded *p* value indicates statistical significance.

^b^ A mixture of EPA and DHA.

When considering only male patients, the most frequently used supplements were multivitamin (24 patients), vitamin D (22 patients), and B‐complex (13 patients); the same top three as the overall sample, but multivitamin was used more frequently than vitamin D. When considering only female patients, the most frequently used supplements were vitamin D (49 patients), multivitamin (38 patients), and calcium (29 patients). Among the female patients, the top three included calcium instead of a vitamin B‐complex, which was in the top three overall and for males only. Females were significantly more likely to be taking calcium, vitamin B12, and magnesium compared to males (Table 2).

Associations between supplement use and patient characteristics were analyzed using chi‐square tests (Table 3). There was no association (*p* = .091) between supplement use and sex, meaning that neither males, nor females were more likely to use supplements than the other. There was an association (*p* < .05) between supplement use and the patients' smoking status, with nonsmokers more likely to use supplements. There was an association (*p* < .05) between supplement use and age with older adults more likely to use supplements. The association persisted (*p* < .05) when the number of supplements a patient reported taking and age were tested as older individuals used a higher number of supplements. The association between number of supplements used and sex was also significant (*p* < .05), with females being more likely to be taking four or more supplements.

**TABLE 3:** Clinical takeaways: Associations between supplement use and demographic factors using chi‐square analyses

Association	Interpretation	*p* value*
Supplement use and sex	Neither sex is more likely to use supplements than the other.	*p* = .091
Number of supplements and sex	Females were more likely than males to take multiple supplements.	***p* < .05**
Supplement use and age	Older adults were more likely to use supplements, specifically the 51–70 year age group and the >70 years age group.	***p* < .05**
Number of supplements and age	Older adults were more likely to take multiple supplements. In the 51–70 and > 70 years age groups, more patients took ≥4 supplements than expected.	***p* < .05**
Supplement use and smoking status	Nonsmokers are more likely to use supplements. It should be noted that the sample of smokers in the study population was small and may have skewed results.	***p* < .05**

^*^ Bolded *p* value indicates statistical significance.

## 4 DISCUSSION

Dietary supplements are being used by over 64.7% of patients in their daily life, prior to, and while receiving periodontal treatment. This is greater than the national average use for adults aged 19 years and older, which was 47.3%, based on the nationwide 2015 Canadian Community Health Survey (CCHS) (Statistics Canada, 2017). The difference between the study population and the Canadian average might be attributed to socioeconomic status. Based on findings from the 2004 CCHS, individuals from a household that reported a higher income were more likely to report using vitamin/mineral supplements (Vatanparast, Adolphe, & Whiting, 2010). In Canada, dental care is not publicly funded; therefore, most Canadians pay for dental treatment either through private insurance (often this is employment‐based) or out‐of‐pocket (Canadian Health Measures Survey results, 2010). Those from a higher‐income bracket are more likely to have private insurance than those from a lower income bracket (Canadian Health Measures Survey results, 2010). Moreover, individuals from Ontario with low household income or no private insurance were more likely to report irregular dental visits or only visiting for dental emergencies (Zangiabadi, Costanian, & Tamim, 2017). Considering that both supplement use and regular dental care appointments are higher among those from households with higher incomes, it is possible that a higher socioeconomic status could explain why the participants in this study reported higher supplement use than the national average.

Higher use among older individuals, particularly for vitamin D, and multivitamin, is consistent with the literature, due to the perceived health benefits such as preventing or reducing risk of chronic disease development (Gahche, Bailey, Potischman, & Dwyer, 2017; McCormack, Mai, & Chen, 2017). Vitamin D is commonly prescribed for bone health and in combination with anti‐osteoporotic medications to prevent fracture for males and females (Dawson‐Hughes et al., 2010). No association was found between dietary supplement use and sex. While this finding differs from the Canadian population where it was found that females are more likely to take dietary supplements than males (CCHS 2015), the current finding may be attributable to age differences of the study populations. As males get older, they could be more likely to use supplements for various health goals (Bailey, Gahche, Miller, Thomas, & Dwyer, 2013).

Females were more likely to take multiple different dietary supplements than males. This could be because they are taking supplements to address multiple health concerns. Therefore, females might be taking supplements “for bone health” and “for healthy joints, to prevent arthritis,” as an example, while a male counterpart might only be taking a supplement “for heart health, to lower cholesterol” (Bailey et al., 2013). An alternative explanation for why females are more likely to take multiple different supplements could be they take more than one supplement to address their primary health concern. For example, as reported in NHANES 2007–2010, females commonly reported taking supplements to support bone health; this could mean they take vitamin D, calcium, and a multivitamin, whereas a male may solely use a multivitamin to support his health (Bailey et al., 2013). Higher supplement use was also associated with being a nonsmoker. While this result must be interpreted with caution because there were only 13 patients (6%) who were smokers, this finding is supported by previous reports (Bailey et al., 2013; Johnston, Fritz, & Ward, 2013) including data from NHANES 2007–2010 which showed that nonsmokers report being supplement users more frequently than smokers in the general population (Bailey et al., 2013).

A limitation of the current study is that patients reported the supplements they were taking at their first appointment only; data on whole food intake were not collected, nor were biological samples collected to measure the levels of the different supplements in the blood. In particular, baseline intake or status of vitamins and minerals are important to consider prior to supplementation to evaluate if supplementation is simply correcting a deficiency or low status for a nutrient as this may affect the clinical outcomes.

The strengths of this study include the large sample size and all patients were undergoing implant or soft tissue graft surgery; a population that may benefit from supplement use during the healing phase. Moreover, the findings are largely generalizable to the overall adult population as both sexes and a range of adult ages were included. This study also shows that supplement use had remained fairly consistent over time when compared to a similar study previously conducted among this population (Johnston et al., 2013). It was previously reported that 64.1% of patients attending this clinic for general examination or surgical consultation were taking dietary supplements (Johnston et al., 2013). The patients included in this present study—patients undergoing implant or soft tissue graft surgery—are of particular interest because following their surgery, they could be in a position to further support their recovery by adding dietary supplements to their post‐surgical care (Gorecki et al., 2018).

In conclusion, the majority of the study population is already taking dietary supplements on a daily basis. For this reason, it seems feasible to add dietary supplements to a patient's standard care for maintaining periodontal health or to support recovery following periodontal surgery. Given many patients who undergo periodontal procedures already include supplements in their routine, conducting a clinical trial investigating the effect of supplements with antioxidant or anti‐inflammatory activity on healing is highly feasible. It is also important to recognize that a healthy diet plays an important role in achieving and maintaining periodontal health. This is an important message for patients as well as the general public. Moreover, there is interest in the health benefits of plant‐based diets, and that there might specifically be benefits to oral health (Mazur et al., 2020). Future studies could examine if healing is enhanced with a plant‐based diet following periodontal procedures.

## CONFLICT OF INTEREST

The authors declare no conflicts of interest.

## CLINICAL SIGNIFICANCE

Scientific Rationale for the Study: There is evidence that nutrition plays a role in the maintenance and healing of periodontal tissues. The relationship between specific nutrients and healing is less clear. Intervention studies are needed to determine if supplementation of specific nutrients can accelerate healing following periodontal treatment. Before these intervention studies can occur, the acceptability of dietary supplement use among this population must be known.

Principal Findings: On average, this population uses dietary supplements more than the general Canadian population (64.7% vs. 47.3%). Males and females are both likely to use dietary supplements. There is a trend that older adults are more likely to use dietary supplements than younger adults.

Practical Implications: There is a high rate of dietary supplement use among patients undergoing periodontal surgery. It can be inferred that there would be high compliance among this population if a dietary supplement intervention to improve healing post‐surgery was introduced.

## Supporting information


**Appendix** **S1.** Dataset for study.

## Data Availability

The data that supports the findings of this study are available in the supplementary material of this article.
